# Multiple Myeloma Cells Express Key Immunoregulatory Cytokines and Modulate the Monocyte Migratory Response

**DOI:** 10.3389/fmed.2017.00092

**Published:** 2017-06-27

**Authors:** Leonardo Freire-de-Lima, Ana Flávia Fernandes Ribas Nardy, Erivan Schnaider Ramos-Junior, Luciana Conde, Jéssica Santos Lemos, Leonardo Marques da Fonseca, Juliana Echevarria Lima, Angelo Maiolino, Alexandre Morrot

**Affiliations:** ^1^Institute of Biophysics Carlos Chagas Filho, Federal University of Rio de Janeiro, Rio de Janeiro, Brazil; ^2^Institute of Microbiology, Federal University of Rio de Janeiro, Rio de Janeiro, Brazil; ^3^Department of Biomedical Sciences, Arthur A. Dugoni School of Dentistry, University of the Pacific, San Francisco, CA, United States; ^4^Hematology Service, University Hospital Clementino Fraga Filho, Federal University of Rio de Janeiro, Rio de Janeiro, Brazil; ^5^Oswaldo Cruz Institute, Oswaldo Cruz Foundation, Rio de Janeiro, Brazil

**Keywords:** multiple myeloma, monocytes, leukocyte migration, immune response, immunoregulation

## Abstract

Multiple myeloma (MM) is a plasma cell disorder that still remains incurable. The immune dysfunction of the host is a striking characteristic of MM, leading to tumor growth and reducing the survival rate of patients. Monocytes are precursors of conventional dendritic cells (DCs), a major player in the immunity mechanisms driving protective T cell responses against tumor. Herein, we report that human MM RPMI 8226 cell line shows a pronounced chemoattractant activity for monocytes and also expresses enhanced levels of the leukocyte chemotactic cytokines CXCL12, CCL5, MIP-1β, and CXCL10 in association with elevated levels of both key immunoregulatory interleukins such as IL-4 and IL-10. This cytokine profile was observed together with reduced expression of IFN-γ by MM RPMI 8226 cell line, a determinant interleukin involved in the acquisition of cellular-mediated protective responses against tumor cells. We further demonstrate that MM RPMI 8226 cell line expresses elevated levels of soluble form of the intercellular adhesion molecule-1 known to inhibit antitumoral T cell responses. This attractive modulation of immune responses by MM cells might provide a means to impair early antitumor responses during the establishment of cytokine-mediated immunosuppressive tumor niche.

## Introduction

Multiple myeloma (MM) is a B-cell malignancy resulting from aberrant clonal expansion of plasma cells that initiates in the bone marrow (BM); this accounts for approximately 10% of all hematological cancers (1). Cell-to-cell contact and soluble factors provided by bone marrow stromal cells (BMSCs) play an essential role in the proliferation and survival of malignant plasma cells, contributing to the pathogenesis of the disease (2). The broad range of effects elicited by MM over the BM microenvironment is also able to target host immune effectors. During the development of malignancy, MM cells alter BM microenvironment by modulating the proliferation of mesenchymal stroma cells and establishing a tumor niche (3). The cellular mechanisms involved in the progression of myeloma disease also include the contribution of different populations of myeloid cells (4).

The composition and activation of the myeloid cells are finely controlled in the formation of the MM BM niche. It has been shown that during growth of MM cells in the BM, there is an upregulation of the CXCR3 ligand responsible for inducing a restriction of the localization of effector natural killer cells in the tumor microenvironment. This change in the chemokine microenvironment is further pronounced by the CXCL12 downmodulation, which acts as a chemotactic signal responsible for the exit of the effector natural killer cells of BM, thereby ensuring the subversion of antitumor immune responses (5).

In contrast, MM is able to modulate the activation of myeloid cell populations recruited to the tumor microenvironment. This is the case of dendritic cells (DCs) that accumulate in the BM of patients in clinical progression to advanced stages of disease (6). DCs play a key role in tumor immunity given their exceptional capacity to initiate T cell responses against tumor antigens (7). However, during malignancy of MM, the tumor niche modulates the functional properties of BM DCs by downregulating the expression of proteosome subunits in these cells, therefore, evading the protective antitumor immune responses mediated by human leukocyte antigen (HLA) class I-restricted CD8^+^ T cells (6).

The ontogeny of BM DCs have been the subject of extensive studies. A subset of proliferating cells inside the BM called macrophage/dendritic cell progenitor cells (MDPs) give rise to conventional DCs, plasmacytoid DCs, and monocytes. The monocytes also constitute an important source of dendritic cell precursors, differentiating into inflammatory macrophages or monocyte-derived DCs (8). However, the recruitment of myeloid cells and their precursors as well as the modulation of their stimulatory capacity actively depends on the tumor microenvironment and its cytokines. Herein, we sought to characterize the tumor-derived cytokine stimuli from the widely used malignant L363 and RPMI 8226 hematopoietic human cell lines for myeloma models (9), which could account for the immunosuppressive profile of cancer cell niche responsible to impair the normal development of protective immunity during evasion of host surveillance.

## Materials and Methods

### Cell Lines

The human MM RPMI 8226 and L363 cell lines were cultured in RPMI 1640 medium (Sigma-Aldrich) supplemented with 10% FBS (Gibco), 60 mg/mL penicillin, and 100 mg/mL streptomycin (Sigma-Aldrich) and incubated at 37°C in a humidified atmosphere containing 5% CO_2_.

### Monocyte Isolation

For monocytes isolation, 40 mL peripheral blood mononuclear cells were isolated by Ficoll-Paque (GE, USA) density gradient centrifugation, followed by 2 h of plastic adherence performed with 5 × 10^6^ cells. Non-attached cells were washed with PBS at 37°C.

#### Transmigration Assay

Monocyte migratory activity was assessed in the Transwell system using 5 µm pore size Transwell plates (Costar; Corning). The lower chambers contained 500 µL of fresh MM cell line supernatant. Monocytes (0.5 × 10^6^ in 100 µL of RPMI 1640/10% FBS) were added in the upper chambers and incubated for 4 h at 37°C in a 5% CO_2_ humidified atmosphere. By the end of incubation, migrating monocytes were recovered and counted in a flow cytometer (FACSCalibur™ BD).

#### Real-time qRT-PCR

RNA from MM cell lines was isolated using TRIzol, the quantity and purity were assessed by nanophotometer (NanoDrop ND1000), and only samples with the range of 1.9–2.1 for the A260/A280 ratio was considered for further analysis. To cDNA synthesis, 500 µg of total mRNA were reverse-transcribed using the SuperScript TM III Reverse Transcriptase (Invitrogen, Life Technologies) following manufacturer’s instructions. Real-time PCR was performed with the ABI Prism 7900HT Fast Real-Time PCR System instrument (Applied Biosystems) using the qPCR SYBR Green Core Kit (Eurogentec) according to the manufacturer’s instructions. GAPDH was used as endogenous control (reference gene). The relative levels of mRNA expression were determined by the 2^−ΔΔ Cycle Threshold^ (2^−ΔΔCT^) method. Primers used to amplify specific gene products are described as follows: CCL4 F 5′ TGTGCTGATCCCAGTGAATC 3′ R 5′TCAGTTCAGTTCCAGGTCATACA 3′; CCL5 F 5′ CCAGCAGTCGTCTTTGTCAC 3′ R 5′ CTCTGGGTTGGCACACACTT 3′; CXCL9 F 5′ CCAGTAGTGAGAAAGGGTCGC 3′ R 5′ AGGGCTTGGGGCAAATTGTT 3′; CXCL12 F 5′ ATTCTCAACACTCCAAACTGTGC 3′ R 5′ ACTTTAGCTTCGGGTCAATGC 3′.

### Multiplex Cytokine and Chemokine Analysis

In order to measure human cytokines and chemokines, we employed a multiplex array kit (BD Bioscience, USA). The cell lines RPMI 8226 and L363 were seeded in RPMI medium supplemented with 1% Nutridoma (Roche, USA) at a density of 1 × 10^6^ cells/mL, and kept at 37°C/5% CO_2_ for 48–72 h. The culture supernatants were collected and a quantitative analysis for IL-10, IL-4, IL-12, IFN-γ, CCL2/MCP-1, CCL3/MIP-1α, CCL4/MIP-1β, CXCL-10/IP-10, and intercellular adhesion molecule-1 (ICAM-1) was performed. A mix of beads coated with antibodies and biotin conjugated antibodies was added to tubes containing either cytokine standards for concentration curve or cell supernatants. After 2 h and subsequent washing with PBS 0.05% Tween 20 (PBS/T), streptavidin-PE was added to the mix and lead to incubate for an additional hour. The samples were washed with PBS/T, the beads were ressuspended in PBS containing 10% FBS, and analyzed by flow cytometry (BD FACSCalibur, BD Bioscience, USA). Analysis was performed using the appropriate software (FlowCytomix Pro 3.0, BD Bioscience, USA).

### Statistical Analysis

Statistical analyses were performed with GraphPad Prism 5 software. Statistical differences between mean values were evaluated by non-parametric Student’s *t-*test. Results were represented as mean ± SD, and differences between groups were considered statistically significant when *p* ≤ 0.05.

## Results

**Figure 1:**
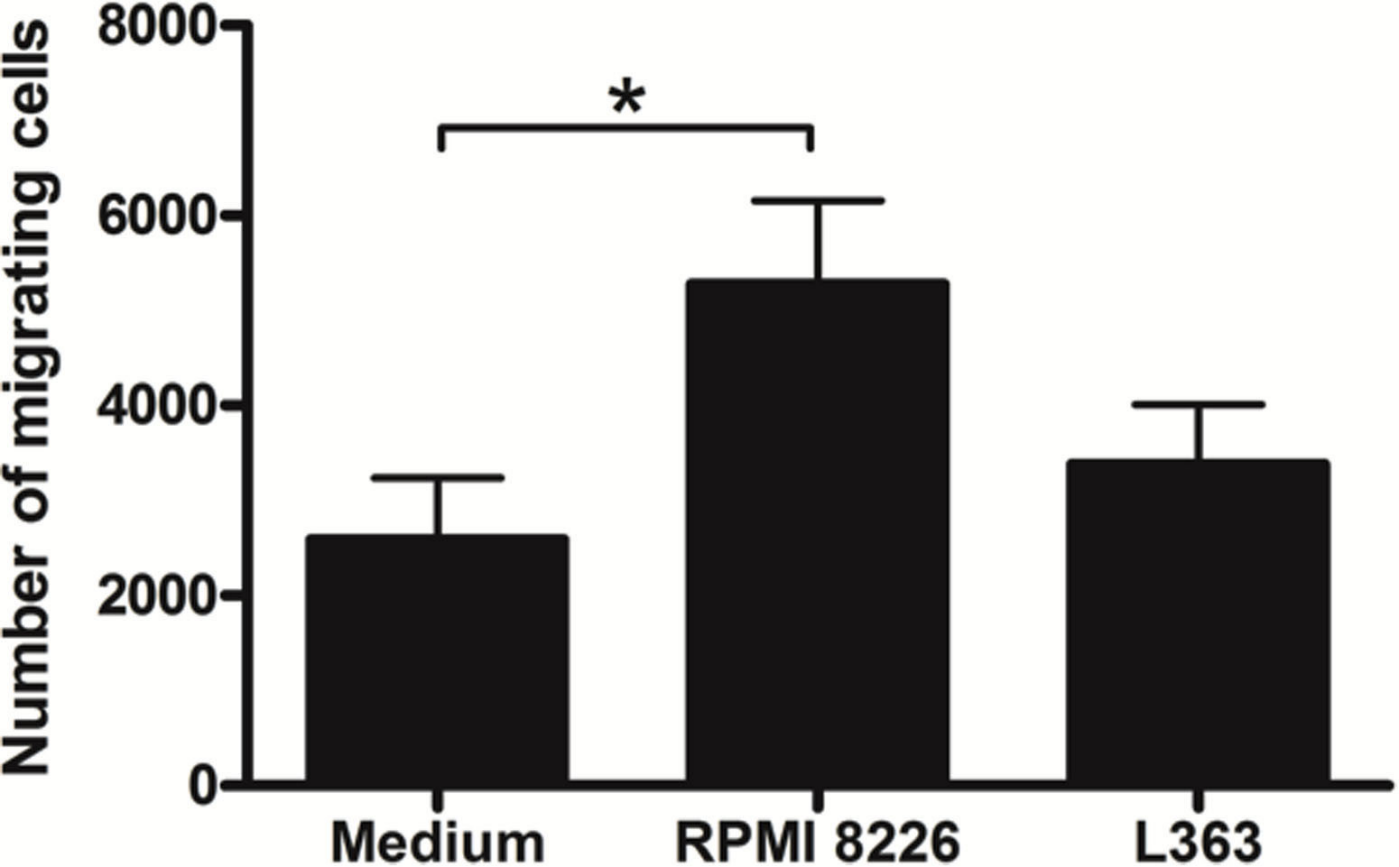
Multiple myeloma (MM) modulates the migratory activity of monocytes. Monocytes (0.5 × 10^6^) isolated from human peripheral blood mononuclear cell were added in the upper chambers and their migratory capacity was assessed in the Transwell system by using 5 µm pore size plates. The lower chambers contained 500 µL of fresh MM cell lines supernatant. After 4 h of migration, cells suspension contained in the lower chambers were recovered and the number of migrant monocytes analyzed by flow cytometry. A greater chemoattractant effect of the RPMI 8226 cell line on the monocyte migration was observed. Bars indicate means ± SDs from five independent experiments.

Common myeloid progenitors give rise to MDPs. These cells subsequently originate DCs by common DC progenitors, as well as monocytes. Monocytes, in turn, can also give rise to DCs both under inflammatory and steady-state conditions. Due to the crucial role played by DCs in tumor immunity, through the initiation of adaptive immune responses mediated by T lymphocytes, we sought to investigate the ability of MM cells to potentially affect the initial stages of DC differentiation, by altering the biological process of their monocytic precursors. During this approach, we performed a monocyte migration assay using the supernatants of two different MM cell lines, RPMI 8226 and L363. Our data show an increase in monocyte migration toward the stimuli induced by RPMI 8226 supernatant (Figure 1).

**Figure 2:**
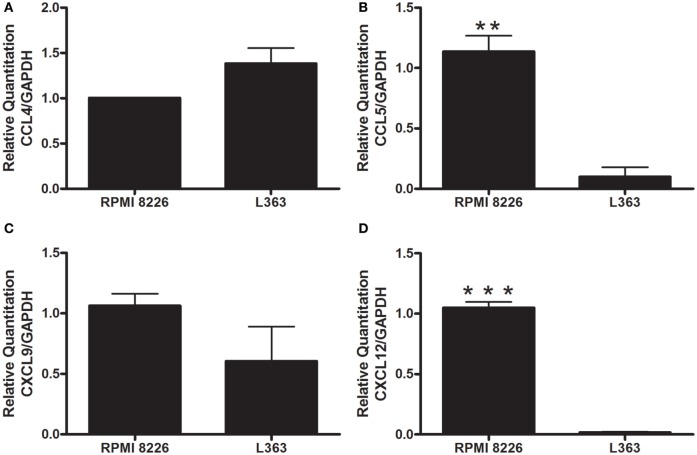
Evaluation of leukocyte chemotactic cytokine expression by human MM cell lines. The levels of CCL4 **(A)**, CCL5 **(B)**, CXCL9 **(C)**, and CXCL12 **(D)** mRNA were analyzed by quantitative real-time PCR. The values were normalized to GAPDH mRNA expression level. The expression of both CXCL12 **(B)** and CCL5 **(C)** were higher in the RPMI 8226 cell line, suggesting a role of these chemokines in the chemoattraction of monocyte cells. All experiments were performed in triplicate and the data shown are representatives of two independent experiments using five mice per group. Indicated differences between groups are significant (**p < 0.01, ***p < 0.001).

**Figure 3:**
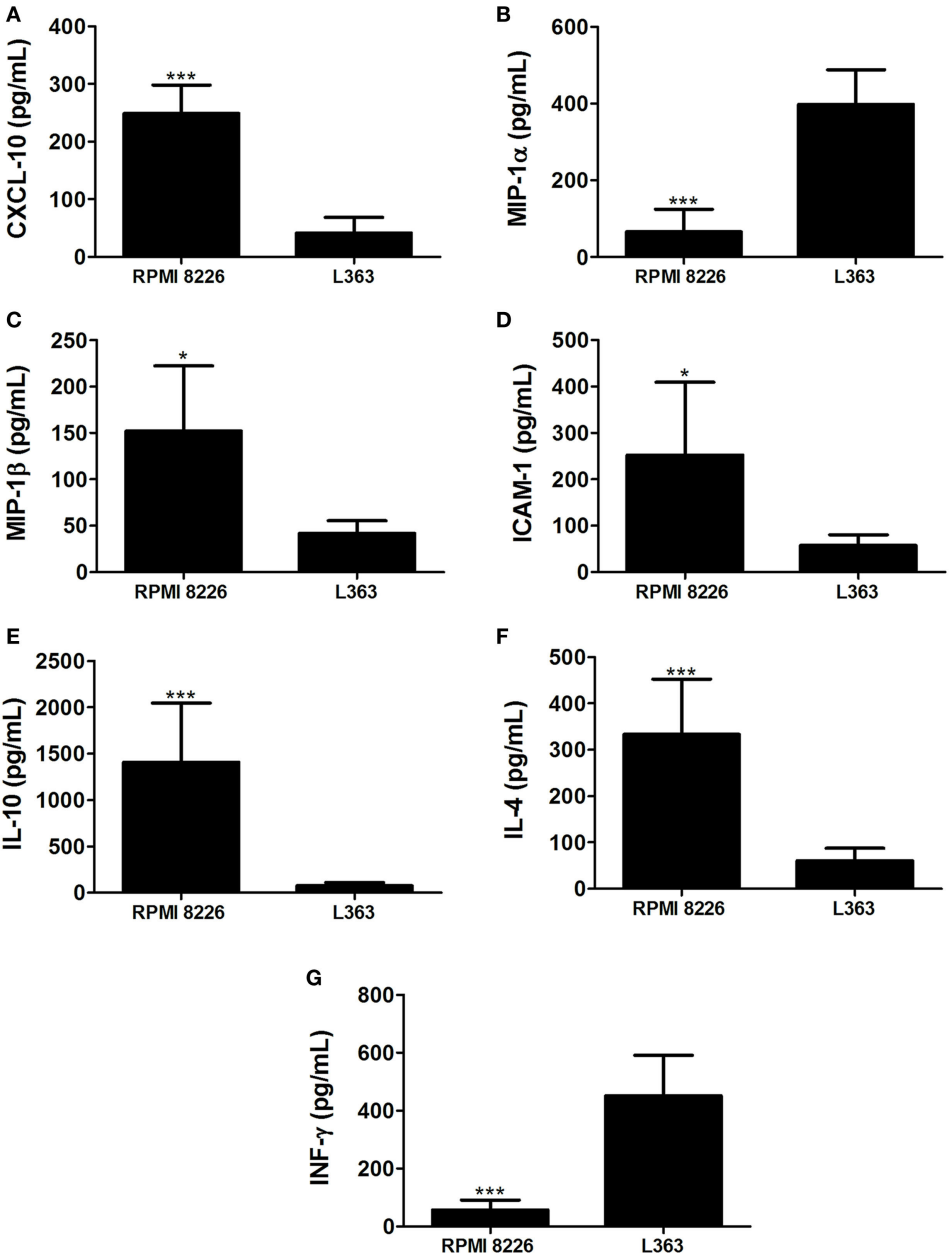
Multiple myeloma (MM)-mediated skewing toward an anti-inflammatory interleukin profile. Levels of chemokines CXCL-10 **(A)**, MIP-1α **(B)**, MIP-1β **(C)**, intercellular adhesion molecule intercellular adhesion molecule-1 (ICAM-1) **(D)**; and the interleukines IL-10 **(E)**, IL-4 **(F)**, and IFN-γ **(G)** were assessed in culture supernatants of both MM RPMI 8226 and L363 cell lines by using a multiplex cytokine quantification assay. Horizontal bars represent the values (picograms per milliliter) as mean responses ± SDs from duplicate cultures at the same cell numbers, of two independent experiments.

These results prompted us to analyze chemokines from the MM cell lines able to induce monocyte recruitment. It is well conceived that the chemokine CCL4 (MIP-1β) is produced by MM cells, as well as CCL5 (RANTES) and CXCL9 (MIG) (10). High expression levels of CXCL12 by malignant plasma cells have also been described (11). Based on this, we performed qRT-PCR and found enhanced expression of mRNA of chemokines known to induce monocytic attraction in RPMI 8226 cell line (Figure 2). Analysis of cytokine protein expression from supernatant cultures, taken at the exponential growth phase of both MM cell lines, at comparable cell numbers, indicated a significant increase in the expression levels of monocyte/leukocyte chemoattractant CXCL-10 and MIP-1β chemokines in RPMI 8226 cell line as compared to L363 cell line (Figure 3). These data could be associated to the migratory events described herein, which were also more pronounced in the RPMI 8226 supernatants. The increased ability of the RPMI 8226 cell line to secrete chemokines and attract monocytes was correlated with their upregulated expression in the levels of intercellular adhesion molecule ICAM-1 (Figure 3D) as well as the anti-inflammatory IL-10 and IL-4 interleukines (Figures 3E,F). This immunoregulatory cytokine profile was further corroborated by the demonstration that RPMI 8226 cell line produced low levels of the pro-inflammatory IFN-γ interleukine (Figure 3G).

## Discussion

Multiple myeloma cells, although also detected in extramedullary locations, are confined in the BM during the intial stages of the disease. The direct relation between MM cells and BM microenvironment, through cell-to-cell contact and soluble factors provided by BMSCs, plays a vital role in the pathogenesis of disease. These interactions also result in osteoclastogenesis and angiogenesis, and activation of signaling pathways responsible for growth, survival, drug resistance, and tumor-cell migration (2). Over the last decade, several biochemical studies have been conducted with human myeloma cell lines to investigate the effects of malignancy in disease progression (12–15).

Genetic studies showed an increase in both number and complexity of the structurally rearranged chromosomes of human myeloma cells, including L363 and RPMI 8226 cell lines. Although such variations were capable of modulating the expression of genes involved in cell cycle control, apoptosis, and proliferation (16), the hypothesis that molecules able to modulate the host immune response may be differentially expressed by such cells, cannot be ruled out. These cell lines show a similar profile as regards to protection of apoptosis-induced cell death mediated by the autocrine loop involving BAFF, APRIL, and their receptors from L363 and RPMI8226 cell lines (17). Analysis of toll-like receptor (TLR) expression at protein level indicated that TLR1, TLR3, TLR4, TLR7, TLR8, and TLR9, were similarly expressed in most human myeloma cell lines, including L363 and RPMI 8226 cells, as well as in primary cells from MM patients (18).

The BM, containing progenitors of both lymphoid and myeloid lineages, promotes vital processes in hematopoiesis. Myeloid cells include granulocytes, macrophages, monocytes, as well as DCs. Monocytes may differentiate into inflammatory macrophages or monocyte-derived DCs (8). It has been shown that, in MM, there is an accumulation of DCs in the BM of patients in clinical progression of disease. These accumulated DCs show a dysfunction in the stimulation of cell-dependent CD8^+^ T antitumor responses due to a downmodulation of the proteosome subunits (6).

The factors responsible for triggering the recruitment and accumulation of non-functional DCs in the BM of patients with advanced forms of MM remain still unknown. Herein, we showed that chemokines such as CCL5, CXCL12, CXCL-10, and MIP-1β are enhanced in the RPMI 8226 MM cell line and these findings may be correlated to the increased monocyte migration toward the stimuli induced by these cells. These chemokine/receptor axes are of extreme importance during monocyte migration in the inflammatory microenvironment (19–21).

Moreover, the CCL5/receptor axis has been described to display several effects in cancer, including the recruitment of immunosuppressive cells like Tregs and monocytes. This chemokine is able to reprogram immature myeloid cells in order to convert them into immunosuppressive MDSCs, attracting them to the tumor niche. Moreover, in MM, it is proposed that CCL5 secreted by tumor cells contributes to disease progression mainly by its action in bone lysis (22). MDSCs comprise a heterogeneous mixture of myeloid cells at different maturation stages with the ability to expand in blood and lymphoid organs of animals and cancer patients (23). In the context of MM, Brimnes et al. observed an increase of both CD14^+^ HLA-DR^low^ MDSCs and Tregs in newly diagnosed patients (24). In this sense, it is conceivable to think that those cells may contribute significantly to the immune dysfunction found in MM patients.

Interestingly, our findings demonstrate that RPMI 8226 MM cell line is able to secrete soluble form of ICAM-1. It has been shown a clinical correlation between the increased levels of circulating ICAM-1 and myeloma progression in untreated patients (25). It is possible that this correlation with disease severity has to do with the action of soluble forms of cell adhesion molecules in inhibiting the interaction of cancer-specific T cells in the tumor microenvironment (26). This evasion mechanism of immune-adaptive responses could contribute to the growth of the MM in the BM, thus potentiating their metastasis to the blood circulatory system and peripheral lymph nodes.

Another aspect of the subversion of anti-myeloma responses in the tumor niche may be due to a probable skewing of the immune responses to an anti-inflammatory profile. In this context, our data show that the RPMI 8226 MM cell line is capable of secreting high levels of IL-10 and IL-4 immunoregulatory cytokines, while expressing decreased levels of IFN-γ interleukin. This cytokine milieu profile could favor the tolerance of T myeloma-specific responses in the tumor microenvironment, thus subverting the immunity. The potential anti-inflammatory action of the tumor microenvironment and the existence of an intrinsic ability of MM cells to attract monocytes by means of secreted factors could also contribute to the dysfunction of DCs during disease (6). Taking into account the potential of monocytes to differentiate into DCs (8), it is possible that MM cells could affect the early stages of DC differentiation, thereby potentially modulating the adaptive immune responses to their own advantage.

There is significant variation in the survival of patients with myeloma that might reflect the differences in the clonality and malignancy of myeloma cells (27–30). In this regard, our findings showing differences in the chemoattractant responses and cytokine expression profiles from human myeloma cell lines might reflect variation in clinical features observed in MM patients. During the past decade, the development of clinical strategies capable of overcoming the immune dysfunction caused by MM has been carried out. Our results suggest a possible role of myeloma cells in dampening protective immune responses. Further evaluation in larger primary tumor samples of patients with differences in disease outcome is needed in order to better delineate the clinical value of our findings to the pathogenesis of MM. The data shown herein may help future searches about the mechanisms involved in the impairment of antitumor responses during early time points of immune cell development, adding new perspectives to the treatment of this important hematologic disorder.

## Ethics Statement

All the protocols for using human cell lines in the study studies were approved by the Research Ethics Committee of Federal University of Rio de Janeiro (MEMO – no. 551/12 – Protocolo de Pesquisa 166/11), in accordance with the terms of the Brazilian and international guidelines for the welfare regulations.

## Author Contributions

AMorrot: conceived and designed the experiments. LF-L, AN, ER, LC, JL, and LF: performed the experiments. LF-L, AN, JE-L, and AMarrot: analyzed the data and helped evaluate the manuscript. LF-L, AMaiolino, and AMorrot: contributed reagents/materials/analysis tools. LF-L, AN, and AMorrot: wrote the paper.

## Conflict of Interest Statement

The authors declare that the research was conducted in the absence of any commercial or financial relationships that could be construed as a potential conflict of interest.
